# Behavioural addictions as risk factors for incidence and reoccurrence of suicide ideation and attempts in a prospective cohort study among young swiss men

**DOI:** 10.1192/j.eurpsy.2021.469

**Published:** 2021-08-13

**Authors:** M. Wicki, M. Andronicos, J. Studer, S. Marmet, G. Gmel

**Affiliations:** 1 Addiction Medicine, Lausanne University Hospital and University of Lausanne, Lausanne, Switzerland; 2 Institute For Mental Health Policy Research, Centre for Addiction and Mental Health, Toronto, Canada; 3 Frenchay Campus, University of the West of England, Bristol, United Kingdom; 4 Research Institute, Addiction Switzerland, Lausanne, Switzerland

**Keywords:** suicide ideation and attempts, cohort study, addictive behaviours, Depression

## Abstract

**Introduction:**

Substance use disorder, depression and sexual minority are well documented risk factors for suicidal behaviour, far less is known about behavioural addictions.

**Objectives:**

First, to explore associations between behavioural addictions (gaming, gambling, cybersex, internet, smartphone, work) at age 25 and the incidence and reoccurrence of suicide ideation (SID), suicide attempts (SAT), and suicide attempts among those with suicide ideation (SATID) at age 28. Second, to test whether these associations were impacted by adjusting for cannabis and alcohol use disorder, nicotine dependence, sexual orientation and depression.

**Methods:**

Based on two waves of a prospective cohort study of 5,428 young Swiss men, nested models with and without adjustment for risk factors were used to regress SID, SAT and SATID on preceding behavioural addictions.

**Results:**

Without adjustment, each of the behavioural addictions at age 25 significantly predicted the incidence of SID and SAT at age 28. Gambling and cybersex addiction furthermore predicted SATID. When adjusting for other risk factors, associations with behavioural addictions were reduced, whereas depression and cannabis use disorder were the most important and consistent predictors for the incidence and recurrence of SID, SAT and SATID.

**Conclusions:**

Among young Swiss men, behavioural addictions are important predictors of SID and SAT, however a large part of their association is shared with depression and cannabis use disorder. Treatment for addictive behaviors, especially cannabis use can open the door to larger mental health screening and targeted intervention. Crisis intervention among men presenting addictive behaviours with or without substance may therefore be key to preventing suicidal behaviour.

**Disclosure:**

No significant relationships.

